# Real-world experience of switching to taliglucerase among patients with Gaucher disease in Québec: A case series

**DOI:** 10.1016/j.ymgmr.2025.101257

**Published:** 2025-09-24

**Authors:** Angelo Rizzolo, Marie-Claude Miron, Jean-François Delisle, Nathalie Alos, Philippe M. Campeau, François Mercier, Gaucher Disease Program of Québec, Claude Meilleur, Valérie Le François, Pascal Bédard, Luis Ospina, Denis Bérubé, Anne Lortie, Sarit Assouline

**Affiliations:** 1 Division of Hematology, Jewish General Hospital, Montréal, Québec, Canada; 2 Centre Hospitalier Universitaire Sainte-Justine, Research Center, Montréal, Québec, Canada; a Division of Hematology, Jewish General Hospital, Montréal, Québec, Canada; b Centre Hospitalier Universitaire Sainte-Justine, Research Center, Montréal, Québec, Canada

## Abstract

Enzyme replacement therapy (ERT) for Gaucher disease (GD) effectively prevents skeletal, visceral, and hematologic complications of this inherited, lysosomal storage disorder. Taliglucerase is one of the three commercially available ERT products and became the recommended first-line therapy in Québec, Canada in 2016. Thus, 19 patients were switched from imiglucerase to taliglucerase, but more than a quarter experienced significant side effects. Here, we summarize these patients' clinical course and describe 6 suspected product-related adverse-effects.

## 1 Introduction

Gaucher disease (GD) is an autosomal recessive, lysosomal storage disorder characterized by glucocerebrosidase deficiency. This results in intracellular accumulation of glucosylceramide, causing diffuse multisystem inflammation [1]. Enzyme-replacement therapy (ERT) with recombinant glucocerebrosidase is one mainstay of therapy along with substrate reduction therapy (SRT). Three ERT formulations currently exist: taliglucerase, imiglucerase and velaglucerase, however, head-to-head comparisons between them are sparse [2]. Adverse effects of ERT are usually related to intravenous infusion and include headache, skeletal pains, fever, chills and flu-like symptoms. Hypersensitivity reactions can also occur and manifest as rash, pruritus, flushing, nausea/vomiting and diarrhea. Rarely, these can manifest as anaphylaxis [[3], [4], [5]].

Taliglucerase is the most novel ERT product and is produced by expression of recombinant GBA1 in a genetically modified carrot cell line [6]. The advantage of this system is the production of a mature protein with terminal mannose residues, which are required for cellular uptake by macrophages.

In 2016, the single-payer healthcare system in the province of Québec, Canada revised its policy to prioritize taliglucerase as the ERT of choice after an analysis of cost and efficacy by its scientific advisory board, the “Institut national d'excellence en santé et services sociaux [INESS]”. As a result, 19 of our patients were switched from imiglucerase to taliglucerase. Here, we report the real-world experience of taliglucerase in our cohort and highlight six cases of adverse reactions. We wish to raise awareness of the range of symptoms that patients may experience while undergoing this type of switch in ERT.

## 2 Methods

This is a clinician-driven, retrospective case series of patients' experience with ERT in the province of Québec, Canada. The study population consisted of adult and pediatric GD patients, who were treated with imiglucerase and subsequently switched to taliglucerase. Patients who initiated treatment with taliglucerase without prior therapy, untreated patients, and those with missing data were excluded.

Descriptive statistics were used to define the study population and a two-tailed Student's *t*-test was performed to compare the means of GD-related parameters before and after switching ERT formulations. Demographic, clinical, laboratory and imaging data were collected. We defined “pre-switch” values as the last data available prior to the date of the first dose of taliglucerase. We defined “post-switch” values as the first data point available at least 2 years after the first dose of taliglucerase. Three patients were excluded as this data was unavailable. Grading of adverse effects was in accordance with National Cancer Institute Common Terminology Criteria for Adverse Events, version 5.0. All magnetic resonance imaging (MRI) scans were performed at the same academic centre and read by the same radiologist. Given that the bone marrow burden (BMB) score is not validated in the pediatric population, analysis of BMB changes pre and post-switch exclude the two pediatric patients <18 years old. This study was conducted in accordance with the Declaration of Helsinki.

## 3 Results

The Québec provincial GD Program is comprised of 62 patients (9 children, 53 adults), of which 42 are treated. Of the treated patients, 19 were switched from imiglucerase to taliglucerase. None of the patients had received velaglucerase as prior ERT. All switched patients had type 1 GD and two were children <18 years of age. The median age at treatment switch was 30 years. The average duration of treatment prior to switching was 13.6 years in the cohort. Patients were treated with ERT intravenously every 2 weeks as per the product monograph. Hematological, biochemical, and radiographic parameters of GD were similar pre- and post-switch (Table 1, Table 2). Six of the 19 switched patients reported new adverse effects while on taliglucerase, five of whom were switched back to imiglucerase as a result. The following is a description of their clinical course.

**Table 1:** Laboratory and imaging data pre vs post-switch to taliglucerase.

	*N* = 16		
GD Parameter	On Imiglucerase (Pre-Switch)	On Taliglucerase (Post-Switch)	P
Platelet Count x10^9^/L, mean (range)	160 (45–260)	155 (41–248)	0.45
Hemoglobin (g/L), mean (range)	129 (91–161)	127 (81–155)	0.16
Ferritin (ug/L), mean (range)	368 (16–1843)	322 (270–1657)	0.78
Angiotensin converting enzyme (ACE, U/L), mean (range)	73.5 (33–180)	88.6 (18–236)	0.43
BMB Score Spine, mean (range)	4.25 (3.00–5.50)	3.83 (1.00–6.00)	0.43
BMB Score lower extremities, mean (range)	3.14 (0–4.5)	4.45 (2.5–7)	0.03
Liver volume (cm^3^), mean (range)	1586 (804–2235)	1666 (999–3006)	0.68
Spleen volume (cm^3^), mean (range)	676 (107–2669)	812 (117–3579)	0.72

**Table 2:** Laboratory and imaging data pre vs post-switch to taliglucerase, stratified by patients with adverse reactions vs those without reactions.

	N = 16			
GD Parameter	On Imiglucerase (Pre-Switch)		On Taliglucerase (Post-Switch)	
	No Reaction (*N* = 10)	Reaction (N = 6)	No Reaction (N = 10)	Reaction (*N* = 6)
Platelet Count x 10^9^/L, mean (range)	167 (45–218)	168 (86–260)	160 (41–235)	147 (94–248)
Hemoglobin (g/L), mean (range)	127 (91–139)	139 (101–161)	125 (81–149)	136 (116–159)
Ferritin (ug/L), mean (range)	313 (16–1843)	427 (67–1513)	179 (28–555)	548 (83–1657)
Angiotensin converting enzyme (ACE, U/L), mean (range)	72.6 (54–180)	79 (42–142)	86 (45–170)	96 (18–236)
BMB Score Spine, mean (range)	4.25 (3.00–5.50)	4.00 (3.00–4.50)	3.43 (1–5.5)	4.20 (2–6)
BMB Score lower extremities, mean (range)	2.81 (0–4)	3.5 (2–4.5)	4.10 (3–7)	4.50 (2–6)
Liver volume (cm^3^), mean (range)	1510 (804–1960)	1570 (755–2235)	1493 (999–2364)	1847 (1191–3006)
Spleen volume (cm^3^), mean (range)	648 (107–2669)	712 (186–2064)	687 (117–3064)	912 (250–3579)

Patient 1: A Caucasian male with type 1 GD (N370S/L444P genotype) was treated with imiglucerase (30 U/kg/month) from age 10 to 26 due to arthropathy and thrombocytopenia. The patient is known for asthma and multiple food allergies. At age 26, he was switched to taliglucerase (32 U/kg/month) with diphenhydramine pre-medication, but repeatedly experienced infusion reactions characterized by erythema, pruritus, urticaria, and a sensation of nasal congestion, which persisted despite premedication with cetirizine 10 mg and slower infusion rates. After three of such reactions, he was switched back to imiglucerase (30 U/kg/month), which resolved the infusion-related symptoms.

Patient 2: A Caucasian female was diagnosed with type 1 GD (N370S/L444P genotype) at 19 months of age. She never required treatment until 25 years of age due to thrombocytopenia during pregnancy. She received imiglucerase (34 U/kg/month) throughout her pregnancy and the infusion was well tolerated. Planning another pregnancy a year later, she began antepartum taliglucerase (34 U/kg/month). The first infusion without premedication was complicated by nausea and vomiting requiring cessation. The second dose was well tolerated with acetaminophen 1000 mg and cetirizine 10 mg premedication; however, she endorsed fatigue and rhinorrhea lasting for about one week after infusion and self-resolving thereafter. These symptoms recurred upon the subsequent 7 infusions despite acetaminophen 1000 mg and cetirizine 10 mg premedication. She was therefore switched back to imiglucerase (34 U/kg/month) for the remainder of her pregnancy, which she tolerated without adverse effects.

Patient 3: A Caucasian female diagnosed with type 1 GD (L444P/L444P) at 21 months began treatment with imiglucerase (55 U/kg/month) for significant hepatosplenomegaly without neurological or ophthalmologic involvement. She continued imiglucerase without adverse reactions until age 26 years, when she was switched to taliglucerase (59 U/kg/month). Infusions were complicated by nonspecific diffuse abdominal pain and post-infusion nausea, sore throat, and nasal congestion despite premedication with acetaminophen 1000 mg and cetirizine 10 mg. After ∼5 years of treatment with these symptoms occurring sporadically, she developed disabling neck and back pain. Hematologic, radiographic and biochemical markers were stable. She was switched back to imiglucerase (55 U/kg/month) without peri-infusion symptoms, and her pain resolved.

Patient 4: A Caucasian female diagnosed with type 1 GD (N370S/84GG) at 19 years of age began ERT with imiglucerase (60 U/kg/month) for progressive hepatosplenomegaly and thrombocytopenia until switching to taliglucerase (40 U/kg/month) at 48 years of age. Infusions were complicated by low back pain, resolving within 15 minutes after reducing the infusion rate. These episodes recurred infrequently over the next 5 years despite pre-medication with acetaminophen 1000 mg. Despite remaining on taliglucerase (40 U/kg/month) the patient developed clinical, biochemical, and radiographic evidence of GD progression, including worsening back pain, a drop in platelet counts from 86 × 10^9^/L to 34 × 10^9^/L, and qualitatively increased Gaucher-cell infiltration on MRI of the spine and lower extremities. Her dose was thus adjusted to the appropriate dose of 60 U/kg/month without improvement and she was switched back to imiglucerase (60 U/kg/month). Her pain subsided, thrombocytopenia resolved and she resumed normal activities.

Patient 5:A Caucasian male known for trisomy 21 was diagnosed with type 1 GD (N370S/L444P) at 4 years of age. He was started on ERT with imiglucerase (60 U/kg/month) for worsening osteopathy without infusion reactions. He was switched to taliglucerase (61 U/kg/month) at 30 years of age. The patient received his first infusion without premedication, where he endorsed significant nausea and vomiting. Subsequent infusions were administered with diphenhydramine premedication and these symptoms did not recur. However, the patient reported significant disabling fatigue following each infusion lasting “a few days” which persisted for a few months and spontaneously resolved thereafter. He remains on taliglucerase (63 U/kg/month) without reactions at this time.

Patient 6: A Caucasian female was diagnosed with type 1 GD (N370S/84GG) at 12 years of age and was started on ERT with imiglucerase (32 U/kg/month) at 25 years of age for significant bone pains. She was switched to taliglucerase (30 U/kg/month) at 45 years of age. The first and fifth doses were well tolerated, however the second, third and fourth doses were accompanied by a grade 1 infusion reaction manifesting as bilateral shoulder, back and neck pains that subsided with decrease in the infusion rate and acetaminophen 1000 mg premedication. The patient was switched to eliglustat, which was accompanied by severe headaches and finally switched back to imiglucerase (56 U/kg/month) shortly thereafter which is well tolerated.

## 4 Discussion

Our experience of switching to taliglucerase among previously treated patients highlights two clinically relevant points. First, some patients may experience nonspecific yet disabling adverse effects, which can be overlooked– within our cohort, fatigue, muscular pain, or nasopharyngeal congestion were common occurrences. Second, in five cases with persistent symptoms, these resolved after switching back to another ERT, supporting a causal link. In some cases, the change in therapy was delayed due to the non-specific nature of the symptoms and the absence of type 1 hypersensitivity features.

The strengths of the present study are the data available on follow up of 19 patients with GD who were switched from imiglucerase to taliglucerase within our multi-disciplinary program, and its long-term follow-up of at least two years following the switch. Our experience with the switch from imiglucerase to taliglucerase identified that 6/19 (32 %) of our switched patients experienced adverse effects, a frequency higher than those evaluated in the pivotal taliglucerase “switchover trial” [2] where, among the 26 adult patients included, 4 (15 %) experienced infusion-related reactions. However, a direct comparison is limited by three factors: (1) the small number of patients; (2) the definition of an adverse event was more strict in the randomized controlled-trial (i.e. not all reactions were infusional nor typical in our cohort); (3) our study was neither powered nor designed to detect a statistically significant difference in the rate of infusion-related reactions upon switching from ERT formulations. Most challenging in our experience were patients who experienced a nonspecific constellation of functionally limiting symptoms from diffuse bone pains, nausea, vomiting to nasal congestion: while each of these symptoms have been reported [3], the degree of their impact on our patients' quality of life deserves to be highlighted. Given the overlap between the adverse-events described in this cohort with manifestations of GD itself and other etiologies (e.g. viral, musculoskeletal), they may go under-reported by patients, under-recognized by other healthcare professionals, and under-appreciated in the literature. Further studies are required to identify mechanisms and risk factors for the development of treatment-related adverse effects. Finally, this experience highlights the risks of switching therapy for cost-based considerations – here, mandated by our single-payer system – for patients already responding well to one type of ERT. Rather, patients whose GD is stable and are tolerating ERT well, there is no need to switch formulations, and risk new adverse events. Indications to switch ERT formulations should be based on efficacy and tolerance, not cost.

Of note, the efficacy of taliglucerase for treating GD in our cohort aligns with previous reports, with no significant differences in biochemical or radiographic parameters pre- and post-switch [2,3]. Patient 4 experienced worsening GD parameters due to under-dosing upon switching. Interpretation of efficacy is limited by the study's retrospective design and lack of statistical power for efficacy assessment.

It is possible that the development of anti-taliglucerase antibodies may partly explain the adverse events reported in our cohort. Antibodies have been reported among treatment-naïve patients in the PB-06-001 trial and the rate of development appeared to increase over time [7]. The development of these antibodies did not impair the efficacy of the infusion and their association with infusion-related adverse effects is circumstantial at this time. The emergence of anti-taliglucerase antibodies may be more common among previously-treated patients due to cross-reactivity to common epitopes. Compared to imiglucerase and velaglucerase, taliglucerase alfa may be more immunogenic due to its distinct chemical structure, as evidenced by the relatively increased risk of immune-mediated adverse reactions compared to other ERT-formulations [8]. Further studies are required to elucidate the mechanisms of such adverse effects and determine clinical risk factors which may aid clinicians in selecting optimal ERTs for each individual patient. For example, those with underlying severe food allergies (as was the case for patient 1) or those with active autoimmune disease may be at increased risk of immunogenic taliglucerase-related adverse reactions. This remains to be studied in larger cohorts. Limitations of our study include the fact that correlative lyso-GB1 levels and anti-taliglucerase antibody titres are not available for these patients since lyso-GB1 testing was systematically implemented in our program after the switch to taliglucerase and, during clinical follow-up of these patients, the decision to change the product was made regardless of the eventual antibody testing results.

In conclusion, our results highlight that taliglucerase is an effective ERT therapy for GD, however certain patients may experience side-effects. Our real-world experience highlights those cases. Thus, among patients who are stable and tolerating ERT well, our experience suggests that switching them (especially for financial reasons only) may be inappropriate. This report demonstrates the need for awareness and further studies evaluating the mechanisms and risk factors of undesirable treatment complications.

## CRediT authorship contribution statement

**Angelo Rizzolo:** Writing – review & editing, Writing – original draft, Methodology, Formal analysis, Data curation. **Marie-Claude Miron:** Writing – review & editing, Conceptualization. **Jean-François Delisle:** Writing – review & editing. **Nathalie Alos:** Supervision. **Philippe M. Campeau:** Writing – review & editing, Supervision, Conceptualization. **François Mercier:** Writing – review & editing, Supervision, Conceptualization. **Gaucher Disease Program of Québec:** Supervision.

## Declaration of competing interest

None.

## Data Availability

No data was used for the research described in the article.

## References

[1] Stirnemann J., Belmatoug N., Camou F. (2017). A review of Gaucher disease pathophysiology, clinical presentation and treatments. Int. J. Mol. Sci..

[2] Pastores G.M., Petakov M., Giraldo P. (2014). A Phase 3, multicenter, open-label, switchover trial to assess the safety and efficacy of taliglucerase alfa, a plant cell-expressed recombinant human glucocerebrosidase, in adult and pediatric patients with Gaucher disease previously treated with imiglucerase. Blood Cells Mol. Dis..

[3] Zimran A., Wajnrajch M., Hernandez B. (2018). Taliglucerase alfa: safety and efficacy across 6 clinical studies in adults and children with Gaucher disease. Orphanet J. Rare Dis..

[4] Grabowski G.A., Barton N.W., Pastores G. (1995). Enzyme therapy in type 1 Gaucher disease: comparative efficacy of mannose-terminated glucocerebrosidase from natural and recombinant sources. Ann. Intern. Med..

[5] Charrow J. (2009). Enzyme replacement therapy for Gaucher disease. Expert. Opin. Biol. Ther..

[6] Shaaltiel Y., Bartfeld D., Hashmueli S. (2007). Production of glucocerebrosidase with terminal mannose glycans for enzyme replacement therapy of Gaucher’s disease using a plant cell system. Plant Biotechnol. J..

[7] Zimran A., Durán G., Giraldo P. (2019). Long-term efficacy and safety results of taliglucerase alfa through 5years in adult treatment-naïve patients with Gaucher disease. Blood Cells Mol. Dis..

[8] Revel-Vilk S., Szer J., Mehta A. (2018). How we manage Gaucher disease in the era of choices. Br. J. Haematol..

